# Fangchinoline protects against bone loss in OVX mice via inhibiting osteoclast formation, bone resorption and RANKL-induced signaling

**DOI:** 10.7150/ijbs.37162

**Published:** 2020-01-01

**Authors:** Lin Zhou, Guoju Hong, Shangfu Li, Qian Liu, Fangming Song, Jinmin Zhao, Jinbo Yuan, Jennifer Tickner, Jiake Xu

**Affiliations:** 1 School of Biomedical Sciences, The University of Western Australia, Perth, Western Australia, 6009, Australia; 2 Department of Endocrinology, The Fifth Affiliated Hospital, Guangzhou Medical University, Guangzhou, 510700, China.; 3 Orthopedic Department, The First Affiliated Hospital of Guangzhou University of Chinese Medicine, Guangzhou 510006, China.; 4 Department of Spine Surgery, the Third Affiliated Hospital of Sun Yat-sen University, Guangzhou Guangdong, 510630, P. R. China.; 5 Research Centre for Regenerative Medicine and Guangxi Key Laboratory of Regenerative Medicine, Guangxi Medical University, Guangxi, 530021, China

**Keywords:** fangchinoline, RANKL, osteoclast, NF-κB, NFATc1, MAPK

## Abstract

Osteoporosis is a disease characterized by abnormally increased formation and function of osteoclasts. Anti-RANKL treatment using natural medicine is a potential therapy for osteoporosis. Here, we studied the effect of fangchinoline, which is extracted from the root of Stephania tetrandra S. Moore, on osteoclast formation and function. We found that fangchinoline inhibited osteoclastogenesis at doses of 0.5 and 1 µM. In addition, we also examined the mechanism of the inhibitory effect of fangchinoline on osteoclasts. We found that fangchinoline down regulated NFATc1 activity and expression. However, fangchinoline did not affect IκBα degradation and MAPK pathways. In addition, we also found that fangchinoline could protect against bone loss in OVX mice. Taken together, fangchinoline may be a potential compound for osteoporosis.

## Introduction

Bone is a hard tissue that forms the endoskeleton of vertebrates. It is a dynamic tissue that goes through a continuous process of remodelling and renewing to maintain internal homeostasis throughout life 1. Osteoclast-induced bone resorption and osteoblast- induced bone formation are two critical components for this process. Increased numbers and overactivity of osteoclasts is a leading cause of osteoporosis, which is the most common bone disorder in elderly people^2^. Osteoclasts, giant multinucleated cells, are derived from hematopoietic stem cells. The formation and differentiation of osteoclasts requires two key cytokines, receptor activator of nuclear factor kappa-B ligand (RANKL) and macrophage colony-stimulating factor (M-CSF). The interaction of RANKL and its receptor RANK activates a series of signalling pathways including NF-κB, MAPKs and NFAT pathways 3. RANKL- targeted treatment has become a new promising option for osteoclast-related bone diseases.

Fangchinoline is a bisbenzylisoquinoline alkaloid derived from the root of *Menispermaceae* family such as *Stephania tetrandra S. Moore* and *Cyclea peltata Diels* 4. Fangchinoline has numerous pharmacological properties such as anti-inflammatory, anti- oxidant, and neural protection effects 5-7. Tetrandrine, an analogue of fangchinoline, was found to inhibit osteoclast formation and function in cell culture and in a sciatic-neurectomized mouse model 8. However, the role of Fangchinoline in bone resorption and its therapeutic effects on osteoporosis are not known.

In this study, we examined the inhibitory effect of fangchinoline on osteoclast formation, bone resorption. In addition, we investigated the role of fangchinoline in osteoclast marker gene expression and RANKL-induced signalling pathways. Further, we explored the therapeutic potential of fangchinoline using an OVX mouse model. Collectively, we found that fangchinoline protects against bone loss in OVX mice via inhibiting osteoclast formation, bone resorption and RANKL-induced activity.

## Materials and Methods

### Materials

Fangchinoline with a purity ≥ 98% was purchased from Mansite (Chengdu, China). Alpha modified Minimal Essential Medium (α-MEM) and fetal bovine serum (FBS) were purchased from Thermo Fisher Scientific (Scoresby, Australia). Penicillin- Streptomycin and GlutaMAX were purchased from Thermo Fisher Scientific (Scoresby, Australia). The production and purification of recombinant RANKL were described in a previous study 9. Antibodies to IκBα (C21), phosphorylated ERK, phosphorylated P38, NFATc1 (7A6) and β-actin were obtained from Santa Cruz Biotechnology (Paso Robles, CA, USA). Antibodies to P38, phosphorylated JNK and JNK were ordered from Cell signaling (Danvers, MA, USA). Antibody to ERK, MTS and luciferase assay kits were purchased from Promega (Madison, WI, USA). Antibody to v-ATPase-d2 was produced as reported previously 10.

### Osteoclastogenesis assay

Freshly isolated bone marrow macrophages (BMMs) from C57BL/6 mice were plated in T75 flasks and cultured in α-MEM supplemented with M-CSF (50 ng/ml). When cells were confluent, BMMs were seeded in 96-well plates at the concentration of 6 × 10^3^ cells per well. After overnight incubation to allow attachment, cells were differentiated with RANKL (50 ng/ml) and incubated with different concentrations of fangchinoline. Medium was changed every two days for 5 days or until mature osteoclasts were formed. For investigating which stage of osteoclastogenesis is mostly affected by fangchinoline, BMMs were treated with RANKL (50 ng/ml) for 5 days, while 1μM fangchinoline was added to BMM at either day 1, 3 or 5. Then, the cells were fixed in paraformaldehyde for 10 min, followed by three washes with 1 × PBS. After that, the cells were stained with TRAcP staining buffer for counting multinucleated cells and image acquired using a light microscope.

### MTS assay

BMMs were seeded into 96-well plates at the density of 6×10^3^ cells per well and cultured in α-MEM with M-CSF for overnight incubation. BMMs were then incubated with different concentrations of fangchinoline for 48 h. After that, the cells were treated with 20 µl of MTS solution (Promega, Madison, WI, USA) for 2 h, and then absorbance read with a microplate reader (Bio-Rad, Hercules, CA, USA) at 490 nm.

### Hydroxyapatite resorption assay

The effect of fangchinoline on activity of osteoclasts was tested by hydroxyapatite resorption assay. BMMs were seeded in 6-well collagen-coated plates (1 × 10^5^/well) for overnight incubation. Then, the cells were stimulated with RANKL (50ng/ml) for every two days until osteoclasts began to form. After that, the cells were harvested with cell dissociation solution and cultured in hydroxyapatite-coated 96 well plates (Corning, Sullivan Park, NY, USA). Cells were treated with RANKL and fangchinoline for another 48 h. Then, half of the wells were fixed with 2.5 % glutaraldehyde and stained with TRAcP staining buffer for counting the number of multinucleated cells. The remaining wells were bleached with 10% bleaching solution for 10 min and then the images were taken for calculating resorbed area by ImageJ software.

### RNA isolation and analysis

BMMs were seeded in 6-well plates at the density of 1 × 10^5^ per well and stimulated with RANKL (50ng/ml) and treated with fangchinoline in various concentrations for 5 days. Then total RNA was extracted by TRIzol Reagent according to the manufacturer's instructions (Life Technologies, Mulgrave, Australia). Single-stranded cDNA was synthesized from 1μg of RNA using reverse transcriptase with oligo-dT primer. The specific mouse primers used in qPCR reactions were designed as follows: mouse cathepsin K (*Ctsk*) (forward: 5'-GGG AGA AAA ACC TGA AGC-3'; reverse: 5'-ATT CTG GGG ACT CAG AGC-3'), mouse calcitonin receptor (*Calcr*) (forward: 5'-TGG TTG AGG TTG TGC CCA-3'; reverse: 5'-CTC GTG GGT TTG CCT CAT C-3'), V-ATPase-d2 (*Atp6v0d2*) (forward: 5'-GTG AGA CCT TGG AAG ACC TGA A-3'; reverse: 5'-GAG AAA TGT GCT CAG GGG CT-3'), matrix metallopeptidase 9 (*MMP9*) (forward: 5'-CGT GTC TGG AGA TTC GAC TTG A-3'; reverse: 5'-TTG GAA ACT CAC ACG CCA GA-3'), *Nfatc1* (forward: 5'-CAA CGC CCT GAC CAC CGA TAG-3'; reverse: 5'-GGC TGC CTT CCG TCT CAT AGT-3'), TRAcP (*Acp5*) (forward: 5'-TGT GGC CAT CTT TAT GCT-3'; reverse: 5'-GTC ATT TCT TTG GGG CTT-3'), and *Gapdh* (forward: 5'-ACC ACA GTC CAT GCC ATC AC-3'; reverse: 5'-TCC ACC ACC CTG TTG CTG TA-3'). qPCR reactions were performed though ViiA™ 7 Real-time PCR system (Applied Biosystems, Paisley, United Kingdom). All the qPCR reactions were run in triplicates, and normalized by housekeeping gene *Gapdh* and further normalized by control samples.

### NF-κB and NFAT luciferase reporter gene assay

RAW264.7 cells stably transfected with an NF-κB luciferase reporter construct (3қB-Luc-SV40) 11 or with an NFATc1 luciferase reporter construct 12 were used in this experiment to determine the effect of fangchinoline on NF-κB and NFAT activation. Transfected cells were seeded in 48-well plates at the density of 1.5 × 10^5^ cells/well (NF-κB luciferase reporter gene assay) or 5 × 10^4^ cells/well (NFAT luciferase reporter gene assay). After overnight incubation, cells were pre-treated with fangchinoline for 1 h, and then incubated with RANKL (50ng/ml) for 6 h (NF-κB luciferase reporter gene assay) or 24 h (NFAT luciferase reporter gene assay); respectively. Then, the cells were harvested and lysed for measuring luciferase activity using the luciferase assay system (Promega, Sydney, Australia) following the manufacturer's instruction.

### Western blot assays

BMMs cells were seeded in 6-well plates overnight at the density of 1 × 10^6^ cells per well. After 3 h serum starvation, cells were pre-treated with fangchinoline for 1h, then stimulated with RANKL for 0, 10, 20, 30 and 60 min. For long time course western blot assay, cells were cultured in 6-well plates at 1 ×10^5^ cells per well. Fangchinoline was added to the cells on the next day. Then, the cells were stimulated by RANKL at day 1, 3 and 5. Cells were harvested and lysed by RIPA lysis buffer on ice. Protein samples were separated by SDS-polyacrylamide gel electrophoresis (SDS-PAGE) and transferred to nitrocellulose membranes. The membranes were blocked with 5% skimmed milk for at least 1 h at room temperature and then incubated with primary antibodies overnight at 4 ºC. After three times washing with 1 × PBS, membranes were incubated with HRP-conjugated secondary antibodies for 1 h. Proteins on the membranes were visualized by the enhanced chemiluminescence (ECL) system (Amersham Pharmacia Biotech, Sydney, Australia).

### Ovariectomy (OVX) animal model

For OVX experiments, C57BL/6 mice were used in this study. The *in vivo* experiments were conducted according to the protocols proposed by The Guangxi Medical University Ethics Committee [SCXK - (JUN) 2012-0004, China] and the University of Western Australia Animal Ethics Committee. All the mice were raised in standard cages, with the temperature set at 22°C and the lighting condition set at 12 h light and 12 h dark cycle. Mice aged 7-weeks were anesthetized with chloral hydrate and subjected to ovariectomy or sham operation. The ovariectomized (OVX) mice were assigned to four groups, including sham group, OVX group, OVX + fangchinoline (1 mg/ml) group, and OVX + fangchinoline (5 mg/ml) group. Each group contained six mice. In details, after one week to allow recovery from the surgery, OVX mice received intraperitoneal injection of fangchinoline at the concentration of 1 mg/kg and 5 mg/kg every two days. In the meantime, mice from OVX control group and sham operation group were injected with 10% DMSO for comparison. After six-weeks of treatment, all the mice were sacrificed and their femurs were removed for analyzing bone parameters by micro-CT and histomorphometric analysis.

### Micro-CT analysis

The collected femurs were fixed with 4% paraformaldehyde (PFA) for 24 h, followed by three washes with 1 × PBS. Then, samples were scanned by a Skyscan 1176 micro-CT instrument (Bruker microCT, Kartuizersweg, Belgium), using 500 μA source current, 50kV voltage and 0.5mm aluminium filter. Raw images were reconstructed and analyzed using standardized parameters 13 with the accompanied programs (NRecon and CTAn, respectively). The regions of interest were set from 0.5 to 1.5 mm below the bottom of the growth plate. The following trabecular bone parameters were measured: bone volume/tissue volume (BV/TV), trabecular thickness (Tb.Th), trabecular number (Tb.N) and trabecular separation (Tb.Sp).

### Histomorphometric

For histomorphometric study, the mouse femurs were decalcified and embedded with paraffin. The bone samples were sectioned (5 μm thick) and stained by haematoxylin and eosin (H&E) and TRAcP. The stained sections were then scanned with Aperio Scanscope, and analyzed with BIOQUANT OSTEO software (Nashville, USA).

### Statistical analysis

All data demonstrated in this study are representative of one of three or more independent experiments. The data was expressed as mean ± SEM. Statistical significance was determined by paired or unpaired Student's t-tests using Microsoft Excel 2010. *P* value <0.05 was considered to be statistically significant.

## Results

### Fangchinoline inhibits osteoclastogenesis

**Figure 1:**
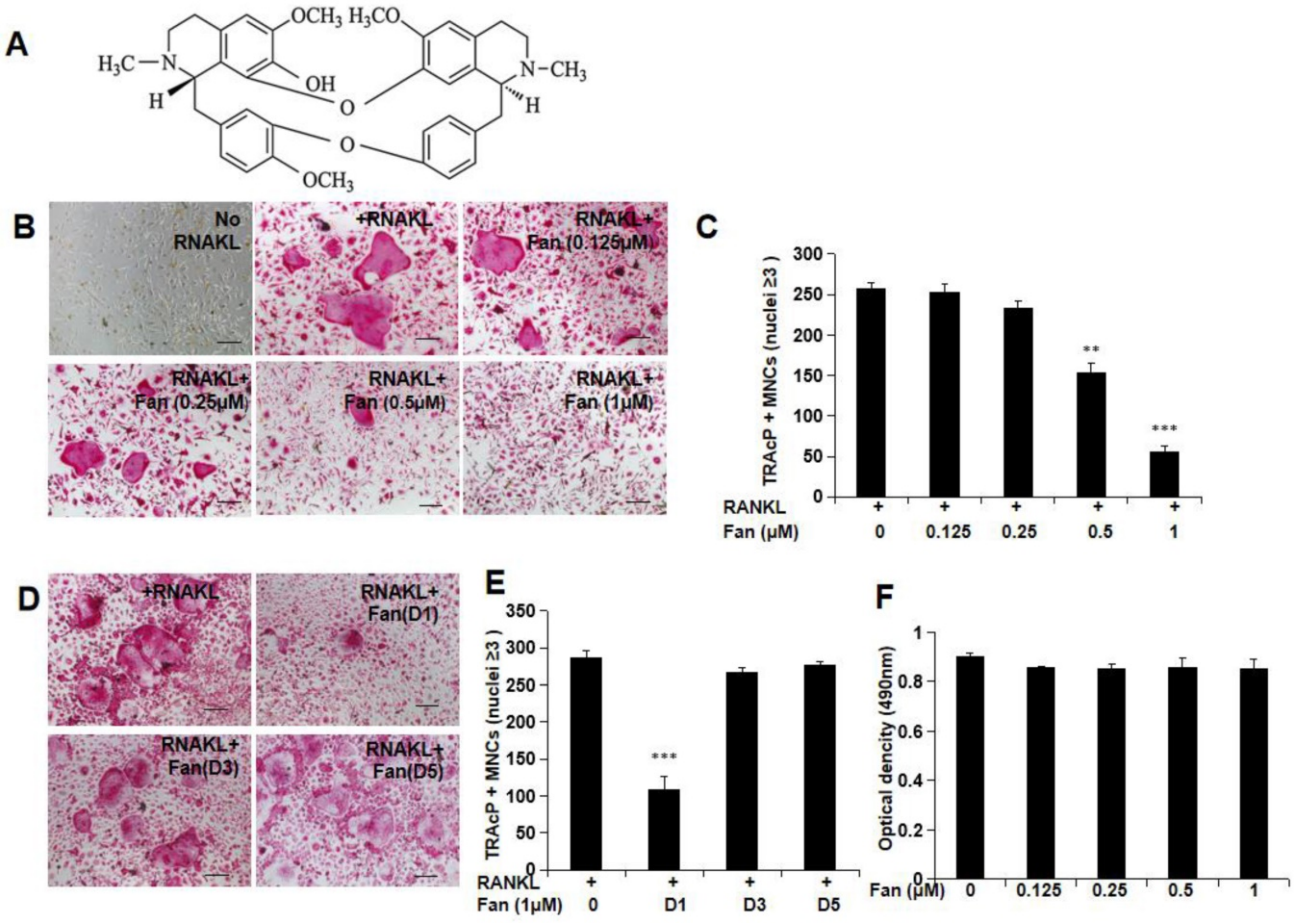
** Osteoclastogenesis and MTS assay.** (A) Chemical structure of fangchinoline. (B) RANKL-induced osteoclastogenesis was suppressed by fangchinoline in a dose dependent manner, which was visualised by light microscope. (scale bars= 100 μm) (C) Consistently, the number of multi-nucleated cells (≥3 nuclei) was significantly decreased from 0.5 μM by counting under optical microscopy. (D) Osteoclast images showed that the treating of fangchinoline at day 1 inhibited osteoclastogenesis. (scale bars= 100 μm) (E) Multi-nucleated cells (≥3 nuclei) counting also showed that fangchinoline inhibited osteoclastogenesis mainly at day 1. (F) MTS results indicated that fangchinoline didn't affect cell viability. ** *P* < 0.01, *** *P*< 0.001 relative to fanchinoline-untreated controls.

To investigate the effect of fangchinoline treatment (Figure 1A) on osteoclast formation, BMM cells were incubated with different concentrations of fangchinoline (0.125, 0.25, 0.5, 1 μM) and RANKL (50 ng/ml) for 5 days. The results suggested that fangchinoline inhibited osteoclastogenesis at 0.5 and 1 μM in a dose-dependent manner (Figure 1B, C). To examine the time course effect of fangchinoline on osteoclastogenesis, 1 μM fangchinoline was added to BMMs for 1, 3 or 5 days in the continuous presence of RANKL (50 ng/ml). Fangchinoline significantly inhibited osteoclastogenesis only when added during the early stages of culture (Figure 1D, E), suggesting that fangchinoline influenced early stages of the osteoclast formation process. MTS results showed that fangchinoline did not impact the viability of BMMs (Figure 1 F), which implied that the inhibitory effect of fangchinoline on osteoclasts was not caused by cytotoxicity.

### Fangchinoline reduces osteoclastic resorption

**Figure 2:**
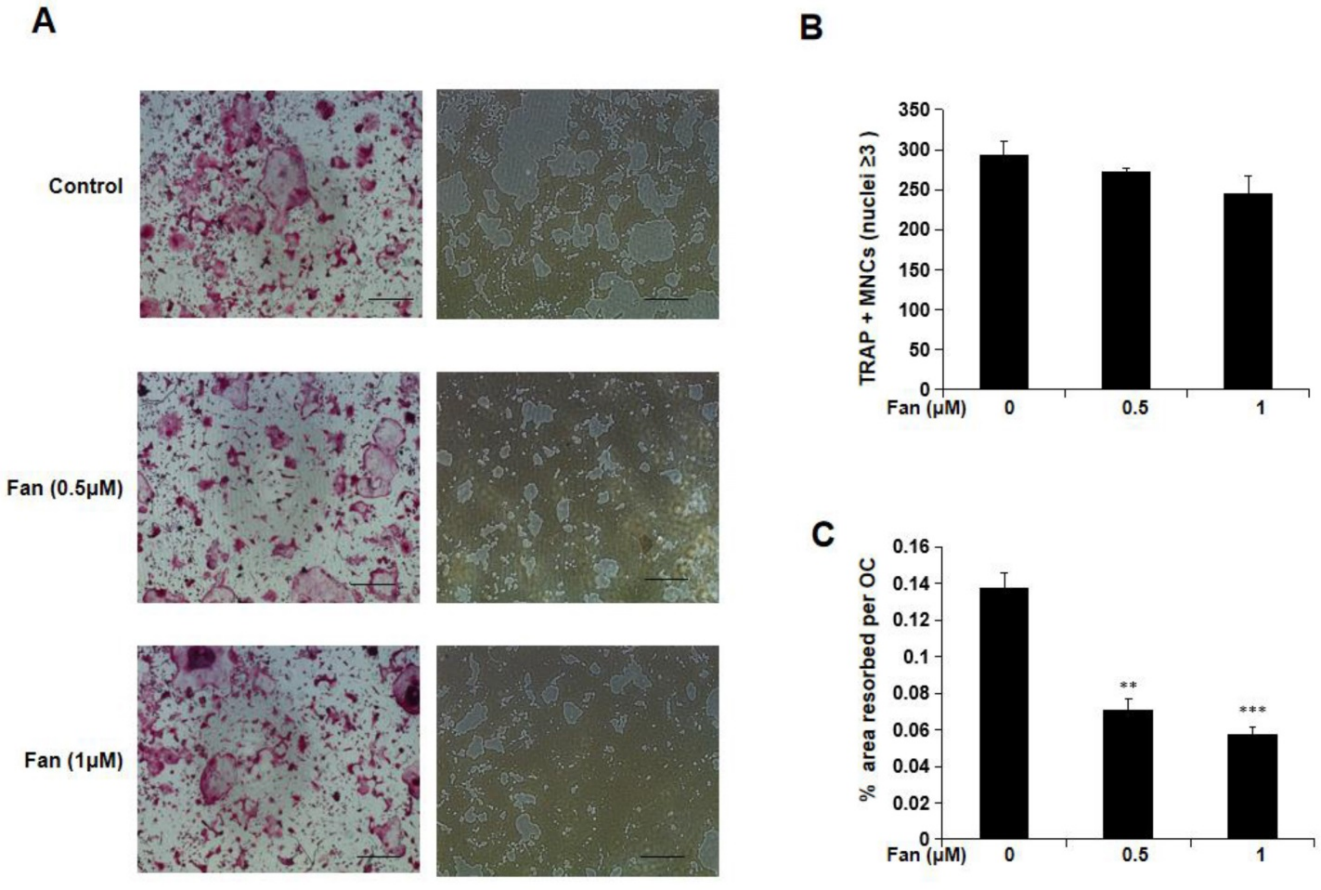
** Resorption assay.** (A) Visualized TRAcP positive multinucleated cells (left side) and resorption pits (right side) on hydroxyapatite-coated plates (scale bars, 500 μm). (B) Fangchinoline didn't affect osteoclast (≥3 nuclei) number. (C) Pits area of per osteoclast was significantly decreased upon the treatment of fangchinoline in a dose-dependent manner on hydroxyapatite-coated plates. ** *P* < 0.01, *** *P* < 0.001 relative to fanchinoline-untreated controls.

To examine the effect of fangchinoline on osteoclasts function, hydroxyapatite-coated 96-well plates were used. Mature osteoclasts were incubated with fangchinoline (0.5 and 1 μM) on hydroxyapatite-coated 96-well plates for 48 h, and the area resorbed per osteoclast was determined. From light microscopy, it was visible that hydroxyapatite resorption area was reduced by fangchinoline (Figure 2A). Consistently, the resorption area per osteoclast analysed by Image J was reduced by fangchinoline, while the osteoclast number was not significantly affected (Figure 2B, C).

### Fangchinoline suppresses the expression of osteoclast marker genes

**Figure 3:**
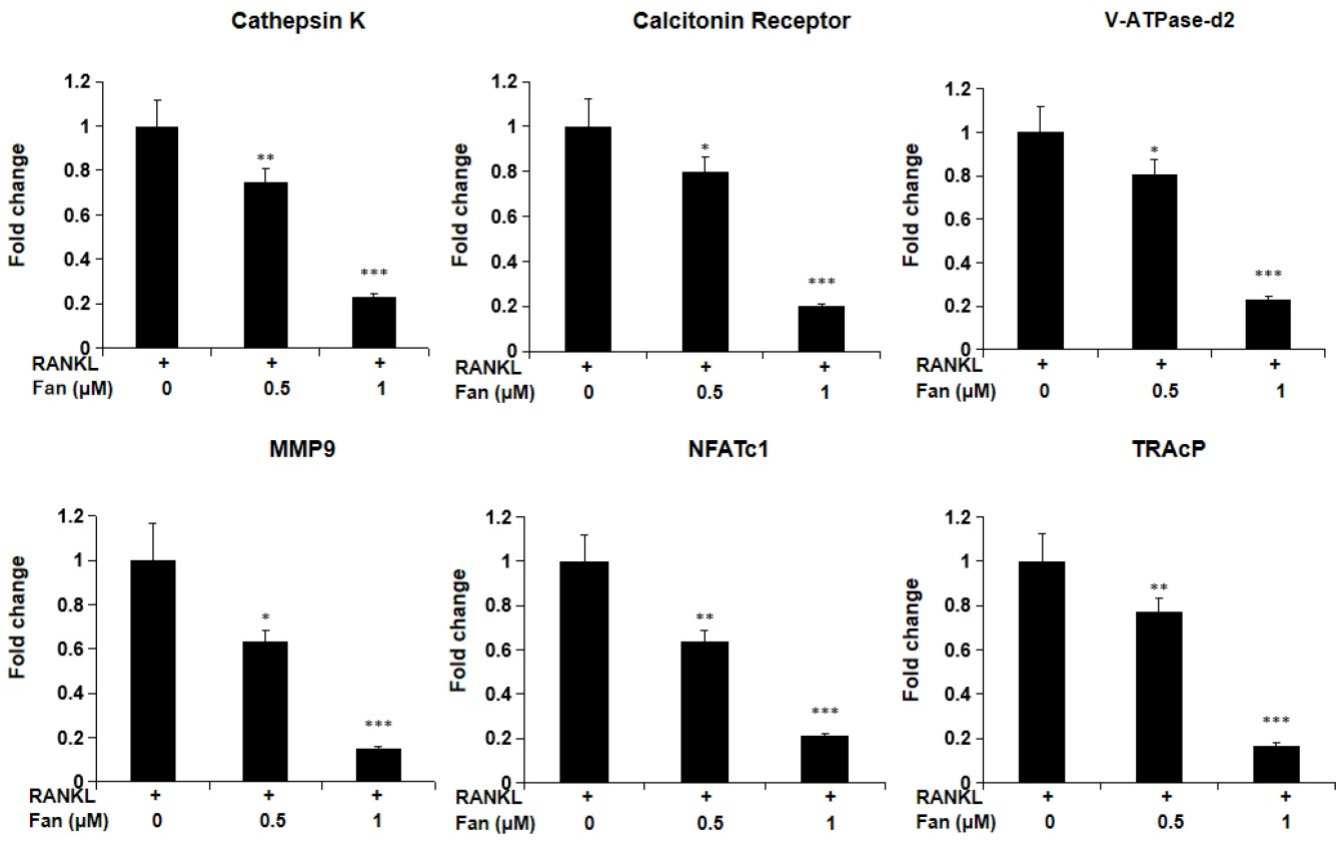
** qPCR assay for osteoclast marker genes.** BMMs were cultured with fangchinoline and RANKL for 5 days. Then, total RNA was extracted for cDNA transcription and qPCR was performed for determining the expression of osteoclast marker genes. The results showed that fangchinoline significantly inhibited the gene levels of *Ctsk* (cathepsin K), *Calcr* (calcitonin receptor), *Atp6v0d2* (v-ATPase-d2), *Mmp9* (matrix metallopeptidase 9), *Nfatc1* (nuclear factor of activated T cells 1) and *Acp5* (TRAcP). * *P* < 0.05, ** *P* < 0.01, *** *P* < 0.001 relative to fanchinoline-untreated controls.

To investigate fangchinoline on RANKL- induced gene expression in osteoclasts, qPCR was employed. As shown in Figure 3, fangchinoline dose dependently reduced RANKL-induced osteoclast marker genes, including *Ctsk*, *Calcr*, *Atp6v0d2*, *Mmp9*, *Nfatc1* and *Acp5*. This result is consistent with inhibitory effect of fangchinoline on the osteoclast formation and function.

### Fangchinoline inhibits NF-κB activity, but not IKBα degradation and the MAPK pathway

**Figure 4:**
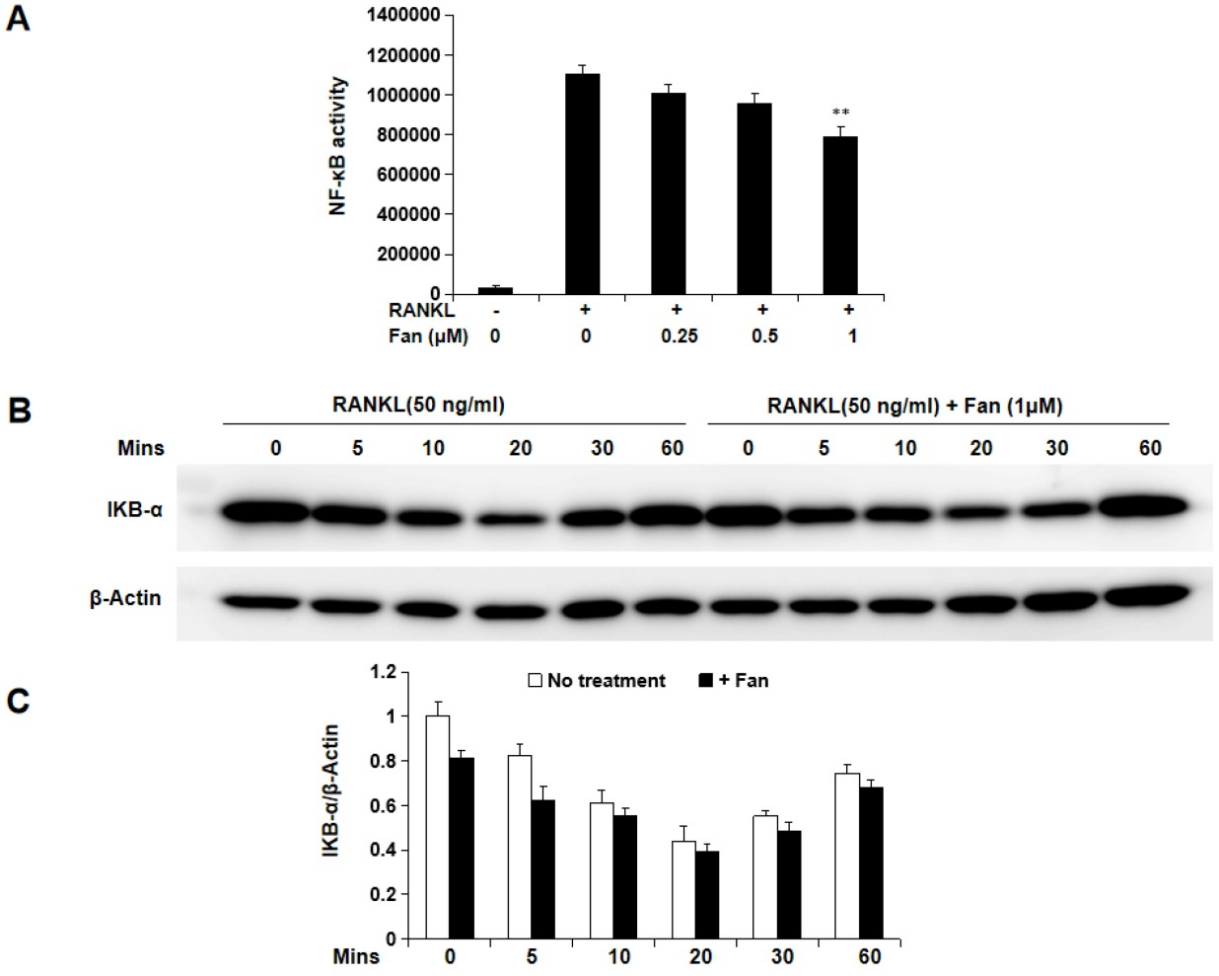
** Fangchinoline suppresses RANKL-induced NF-κB activity, but not IκBα degradation.** (A) NF-κB luciferase assay results suggested that fangchinoline reduced NF-κB activity at 1 μM. (B) BMM cells were pre-treated with fangchinoline for 1 h, then stimulated by RANKL at different time points (0, 5, 10, 20, 30 60 min), followed by harvested for WB assay. (C) The ratios of the density of IκB-α bands relative to β-actin bands were determined using Image J. n=3. The results showed that IκBα degradation was not affected by fangchinoline. ** *P* < 0.01 relative to fanchinoline-untreated controls.

**Figure 5:**
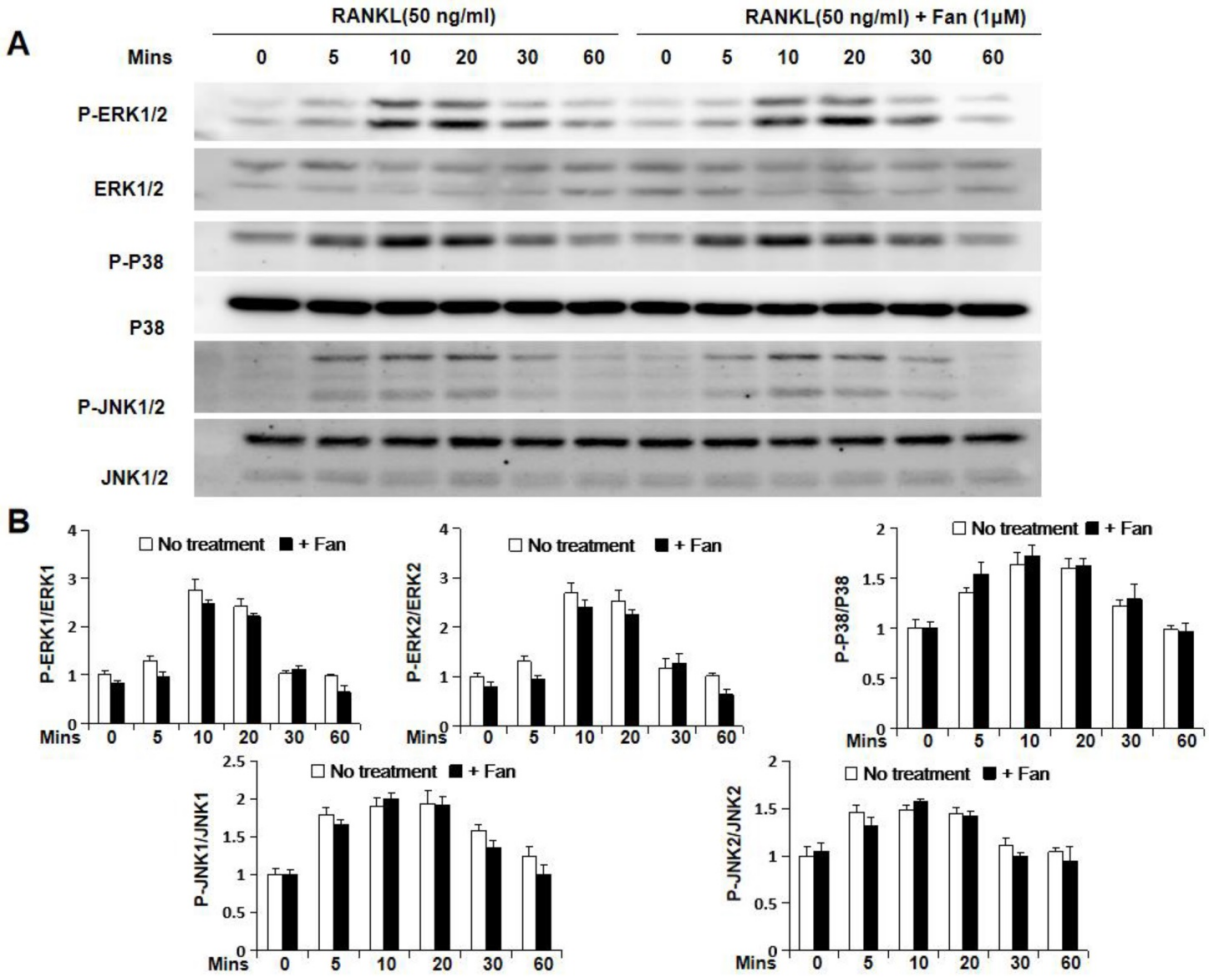
** Fangchinoline doesn't affect RANKL-induced MAPK pathway.** (A) Cells for detecting the effect of fangchinoline on MAPK pathway by WB assay were treated and harvested same as WB assay for assessing IκBα degradation. (B) The ratios of the density of P-ERK1/P-ERK2 bands relative to ERK1/ ERK2, P-P38 bands relative to P38 bands and P-JNK1/P-JNK2 bands relative to JNK1/JNK2 bands were determined using Image J. n=3. The results showed that fangchinoline had no effect on the phosphorylation of ERK1/2, P38 and JNK1/2, indicating the inhibitory fangchinoline on osteoclastogenesis was not through MAPK pathway. The results showed that IκBα degradation was not affected by fangchinoline.

To further study the molecular mechanism by which fangchinoline inhibits osteoclastogenesis, luciferase and western blot assays were used. Luciferase assay showed that fangchinoline inhibits NF-κB activity at the dose of 1 μM (Figure 4A). However, western blot results showed that IκBα degradation was not affected by fangchinoline, suggesting that NF-κB activity was affected downstream of IκBα (Figure 4B). In addition, fangchinoline did not inhibit RANKL-induced phosphorylation of ERK1/2, P38 and JNK1/2 (Figure 5).

### Fangchinoline inhibits NFAT activity, and protein expression of NFATc1 and V-ATPase-d2

**Figure 6:**
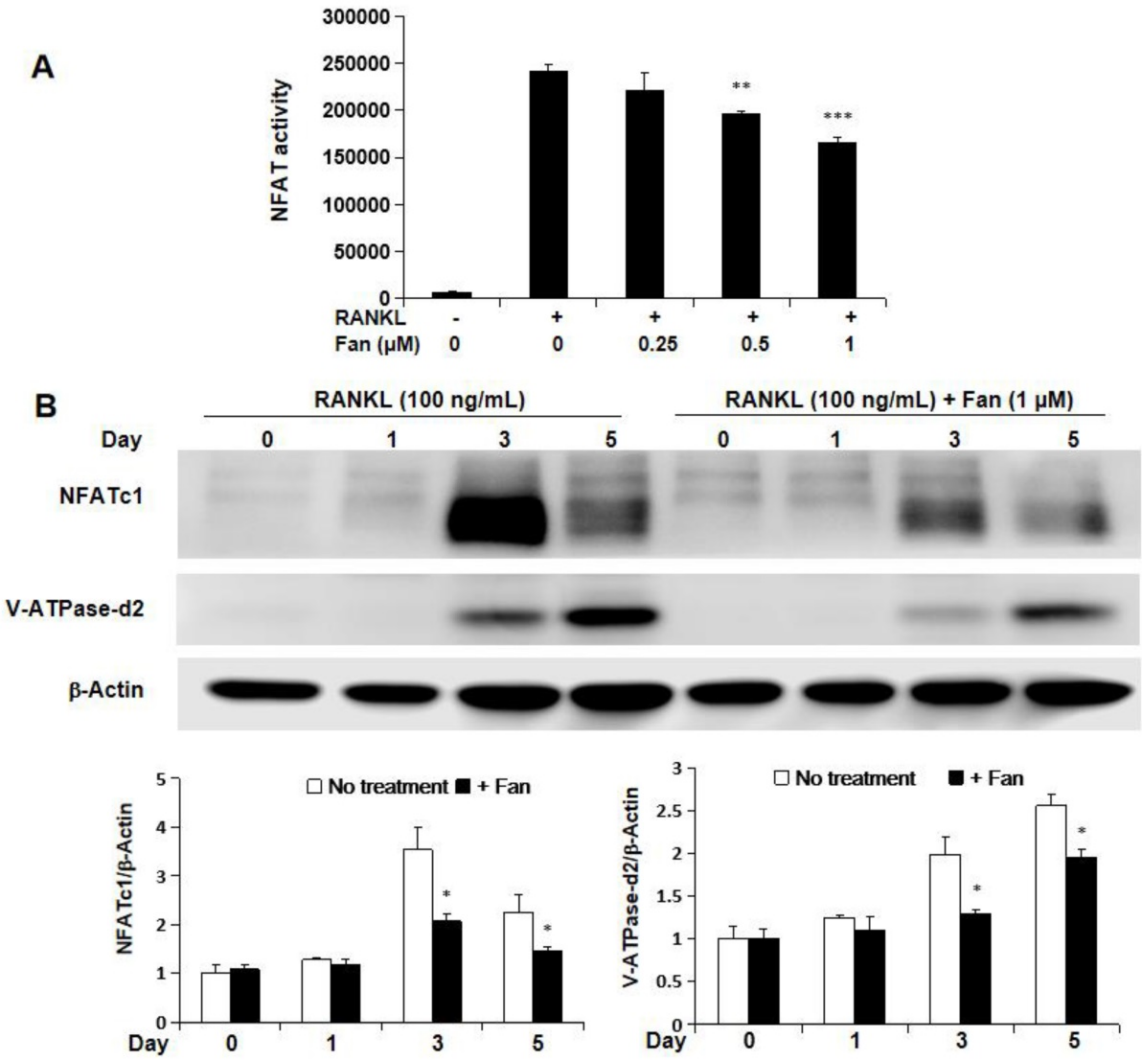
** Fangchinoline inhibits NFAT activity, as wells as the expression of NFATc1 and v-ATPase-d2.** (A) NFAT luciferase results showed that fangchinoline inhibited NFAT activity from 0.5 μM in a dose dependent manner. (B) BMMs were treated with fangchinoline for 5 days and stimulated with RANKL at day 1, 3 and 5. Then, WB assay was performed on the cell lysates. (C) The ratios of the density of NFATc1 bands relative to β-actin bands and v-ATPase-d2 relative to β-actin bands were determined using Image J. n=3. It was shown from results that the protein levels of NFATc1 and v-ATPase-d2 were significantly inhibited by fangchinoline at day 3 and 5. * *P* < 0.05, ** *P* < 0.01, *** *P* < 0.001 relative to fanchinoline-untreated controls.

The effect of fangchinoline on NFAT activity was investigated by NFAT luciferase assay. RAW264.7 cells transfected with NFAT luciferase gene reporter construct were pre-treated with fangchinoline for 1 h, and then incubated with RANKL (50 ng/ml) for 24 h. The results showed that RANKL-induced NFAT activity was inhibited by fangchinoline from 0.5 μM in a dose-dependent manner (Figure 6A). Consistently, the expression of NFATc1 protein was also inhibited by fangchinoline as shown by western blot analysis (Figure 6B, C). In addition, V-ATPase-d2, which is induced by NFATc1, was also inhibited by fangchinoline at 1 μM (Figure 6B, C).

### Fangchinoline protects against OVX-induced bone loss

**Figure 7:**
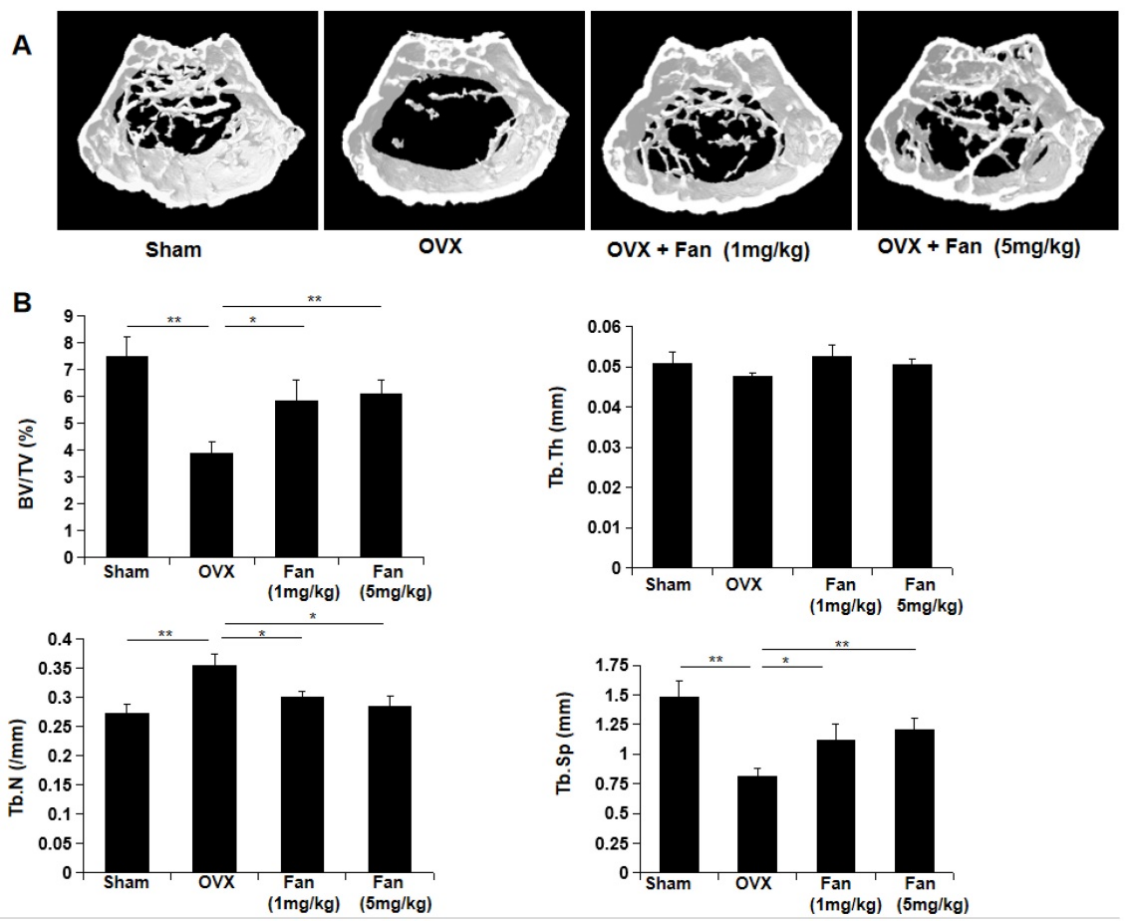
** Fangchinoline protects against ovariectomy-induced bone loss.** (A) Constructed 3D images of proximal femur from sham, OVX, OVX mice injected with low doses and high doses of fangchinoline. (B) The microstructure of bone mass was analysed by following parameters: BV/TV, Tb.Th, Tb.Sp and Tb.N. * *P* < 0.05, ** *P* < 0.01.

To determine the effect of fangchinoline on osteoporosis-induced bone loss, OVX mice which imitate postmenopausal osteoporosis were used in this study. The mice were divided into four groups: sham group, OVX group, OVX + fangchinoline (1mg/kg) group and OVX + fangchinoline (5mg/kg) group. Each group contains 6 mice. Fangchinoline- treated mice had significantly increased bone mass from the 3D images (Figure 7A) and the bone parameters (Figure 7B) as determined by micro-CT. As shown in Figure 7B, fangchinoline-treated OVX mice had a dose dependent increase in BV/TV and Tb.N, and decrease in Tb.Sp. These results indicated that fangchinoline inhibits OVX-induced bone loss in a dose dependent manner.

**Figure 8:**
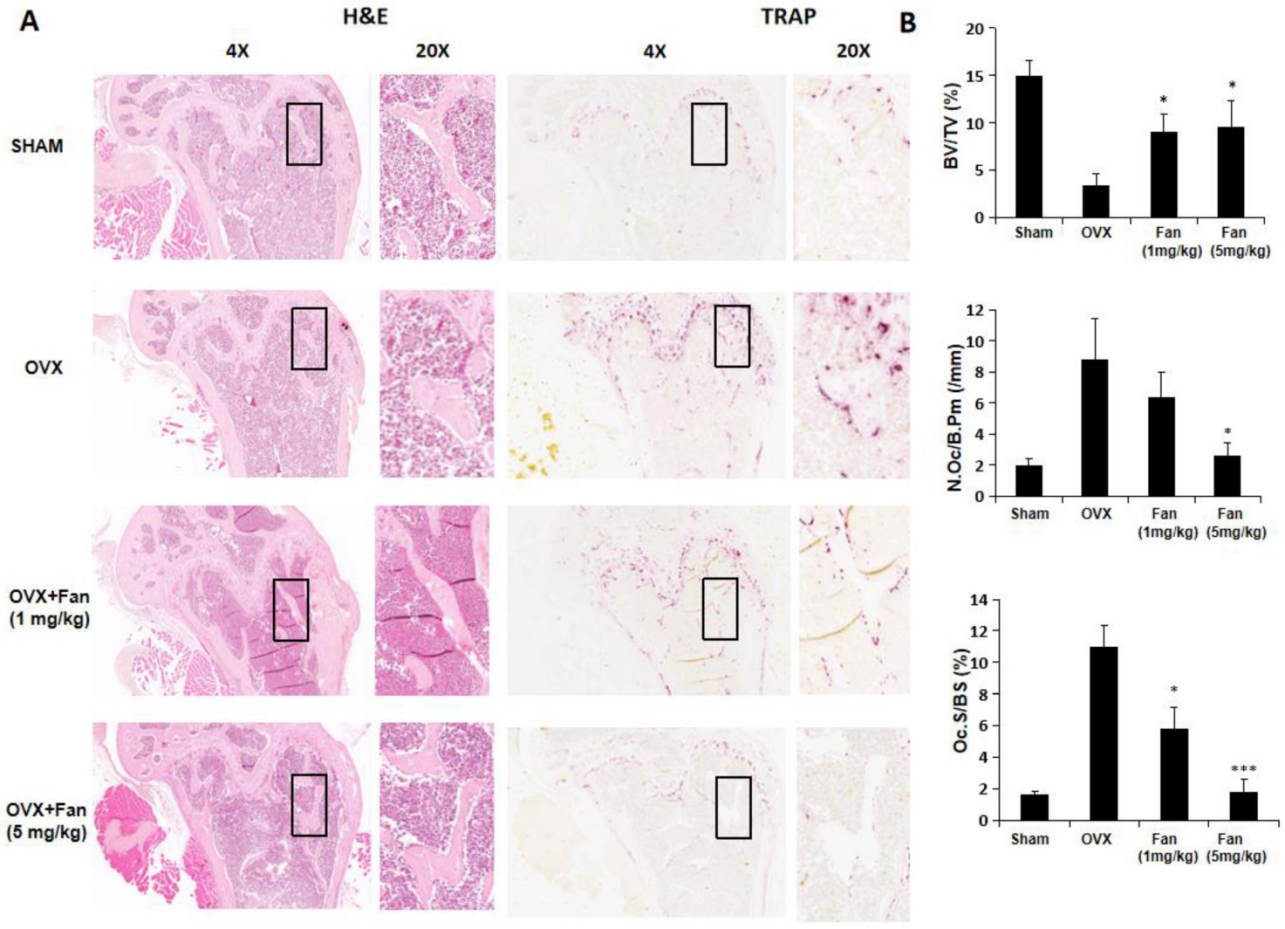
** Fangchinoline protects against ovariectomy-induced bone loss via inhibiting osteoclast activity.** (A) Representative images of decalcified bone stained with H&E and TRAcP from sham, OVX, OVX + fangchinoline (1 mg/ml), OVX + fangchinoline (5 mg/ml). (B) Quantitative analysis of bone volume/total volume (BV/TV), osteoclast surface/bone surface (Oc.S/BS), and osteoclast number/bone surface (N.Oc/BS). n=3. * P <0.05, *** *P* < 0.001 relative to OVX untreated controls.

Histology results further confirmed the protective effects of fangchinoline on osteoporosis- induced bone loss. As shown in Figure 8, BV/TV was significantly increased in fangchinoline treated OVX group compared with vehicle-treated OVX group. In addition, fangchinoline treated OVX group exhibited a significant reduction in osteoclast number/bone surface (N.Oc/Bs) and osteoclast surface/bone surface (Oc.S/BS) compared with vehicle-treated OVX group (Figure 8A, B), suggesting that fangchinoline protects against osteoporosis-induced bone loss through attenuating osteoclast activity.

## Discussion

Osteoporosis is a common disease in elderly populations and may reduce life expectancy due to facture 14. The development of osteoporosis is associated with the overproduction and overactivity of osteoclasts 15. Current treatments against osteoporosis have some side effects, such as bisphosphonates-induced osteonecrosis of jaw and estrogen-induced breast cancer 16, 17. Natural compounds may provide an alternative treatment to osteoporosis since they have been considered to be relatively safer than pharmaceutical synthetic chemicals 18. Numerous natural compounds have been found to attenuate osteoporosis based on cell culture and animal experiments 19. In this study, we explored the therapeutic effect of fangchinoline on osteoclastogenesis and OVX-induced bone loss.

Previous studies have reported that fangchinoline has extensive pharmacological properties including anti-inflammatory 5, anti- oxidant 6, anti-tumour 20, 21, anti-HIV 22 and neural protection effects 23. In this study, we found that fangchinoline suppressed RANKL-induced osteoclast formation and function, and ameliorated bone loss in OVX mice. Our results are in line with previous report which showed that tetrandrine, an analogue of fangchinoline, suppressed RANKL- induced osteoclast differentiation and bone loss in sciatic-neurectomized mice 8.

Osteoclasts, arising from the monocyte- macrophage lineage, are the specific cells, which contribute to bone resorption 24. RANKL and M-CSF are two critical cytokines for the formation and differentiation of osteoclasts. In this study, osteoclastogenesis was dose-dependently inhibited by fangchinoline in the presence of RANKL and M-CSF. MTS results suggested the fangchinoline exerts the inhibitory effect of osteoclastogenesis not through cytotoxicity. Consistently, fangchinoline also down regulated the expression of a series of osteoclasts marker genes, including *Ctsk*, *Calcr*, *Atp6v0d2*, *Mmp9*, *Nfatc1* and *Acp5*.

The inhibitory effect of fangchinoline on osteoclasts function was studied *in vitro* using hydroxyapatite-coated plates, which mimics *in vivo* bone surface to study drug-affected cell functional activity. The hydroxyapatite resorption assay is a simple assay to quantitatively measure the osteoclastic resorbed area. Our results showed that fangchinoline significantly inhibited mature osteoclasts resorption at the concentration of 0.5 and 1 μM, without affecting cell number. Bone resorption is a process that osteoclasts degrade bone and subsequently release calcium to blood 25. The increased bone resorption has critical involvement in the development of osteolytic diseases. Thus, the inhibiting effect of fangchinoline on bone resorption is essential for the potential role of treating osteoporosis. The active osteoclasts degrade inorganic mineral, mainly calcium and phosphate, through secreting acid from the ruffled border, and also dissolve organic matrix of bone, primarily type I collagen, by producing cathepsin K and MMPs 25, consistent with our results that fangchinoline down regulates the gene expression of *Ctsk* and *Mmp9*.

The interaction of RANKL and RANK recruits TRAF6, which is an adaptor protein from the TNF receptor-associated factor (TRAF) protein family, initiating a series of TRAF6 downstream signalling cascades, including NF-κB, NFATc1 and MAPK families (ERK, P38, JNK) 26. NF-κB has been a well-known transcription factor required for survival and differentiation of osteoclasts 27, and play an important role of osteolytic bone disease^28^, ^29^. In our study, NF-κB activity was significantly inhibited by fangchinoline, suggesting the inhibitory effect of NF-κB activity contribute to part of the underlining mechanisms through which fangchinoline regulates osteoclastogenesis. However, fangchinoline had little effect on IκBα degradation, implying that fangchinoline inhibits NF-κB activity through other unknown regulatory events. In addition, fangchinoline did not affect the phosphorylation of ERK, P38 and JNK, suggesting that the inhibitory effect of fangchinoline on osteoclasts is not through MAPK signalling. NFATc1, a master transcription factor for osteoclastogenesis, could be induced by RANKL and auto-amplifies its own transcription. NFATc1 transcription is regulated by calcineurin which is a calcium and calmodulin dependent serine and threonine protein phosphatase 30. Our results showed that fangchinoline inhibited NFAT activity and also down regulated the expression of NFATc1 and V-ATPase-d2. V-ATPase-d2 is transactionally regulated by NFATc1 10, and contributes to the fusion of osteoclasts 31.

The therapeutic effect of fangchinoline on *in vivo* bone loss was investigated by using OVX mice. The OVX animal model, which mimics estrogen withdrawal in postmenopausal women, is the most common experimental method for assessing bone microarchitectural structure of postmenopausal osteoporosis in response to drugs. The process of bone resorption at first surpasses bone formation after ovariectomy, causing the decline of bone mass. Before long, bone resorption and formation reach a new balance 32. In our study, fangchinoline protected the decrease of bone mass reflected by following parameters: the increased BV/TV and Tb.N, and decrease Tb.Sp in OVX mice. In addition, bone histomorphometry results revealed that the protective effects of fanchinoline is through inhibiting the osteoclasts activity by the relative parameter such as osteoclast number/ bone surface (N.Oc/BS) and osteoclast surface/ bone surface (Oc.S/BS). Our results are in line with recent findings showing an inhibitory effect of tetrandrine on bone loss 8, 33, and also provide new mechanistic insights of fangchinoline.

In conclusion, fangchinoline attenuated osteoclast formation, function and ameliorated OVX-induced osteoporosis in mice mainly through suppressing RANKL signalling pathways and osteoclast marker genes expression. Our results suggest that fangchinoline, as a natural compound is a potential candidate drug for the therapy of osteoporosis and other osteoclast-mediated bone diseases.
